# Dienogest alone or dienogest combined with estrogens in the treatment of ovarian endometriomas, that is the question. A retrospective cohort study

**DOI:** 10.1007/s00404-023-07125-2

**Published:** 2023-07-12

**Authors:** Simona Del Forno, Benedetta Orsini, Ludovica Verrelli, Martina Caroli, Anna Chiara Aru, Jacopo Lenzi, Diego Raimondo, Alessandro Arena, Giulia Borghese, Roberto Paradisi, Maria Cristina Meriggiola, Renato Seracchioli, Paolo Casadio

**Affiliations:** 1 Division of Gynecology and Human Reproduction Physiopathology, IRCCS Azienda Ospedaliero-Universitaria di Bologna, Via Massarenti, 13, 40138 Bologna, Italy; 2 Department of Medical and Surgical Sciences (DIMEC), University of Bologna, Bologna, Italy; 3 Department of Medical and Surgical Sciences for Mother, Child and Adult, University of Modena and Reggio Emilia, Azienda Ospedaliero-Universitaria Policlinico, Modena, Italy; 4 Department of Biomedical and Neuromotor Sciences, Alma Mater Studiorum - University of Bologna, 40138 Bologna, Italy

**Keywords:** Endometriosis, Medical therapy, Ultrasound, Pelvic pain

## Abstract

**Purpose:**

to compare the effects of Dienogest 2 mg (D) alone or combined with estrogens (D + ethinylestradiol 0.03 mg, D + EE; D + estradiol valerate 1–3 mg, D + EV) in terms of symptoms and endometriotic lesions variations.

**Methods:**

This retrospective study included symptomatic patients in reproductive age with ultrasound diagnosis of ovarian endometriomas. Medical therapy for at least 12 months with D, D + EE or D + EV was required. Women were evaluated at baseline visit (V1) and after 6 (V2) and 12 months (V3) of therapy.

**Results:**

297 patients were enrolled (156 in the D group, 58 in the D + EE group, 83 in the D + EV group). Medical treatment leaded to a significant reduction in size of endometriomas after 12 months, with no differences between the three groups. When comparing D and D + EE/D + EV groups, a significant decrease of dysmenorrhea was detected in the D group than in D + EE/D + EV group. Conversely, the reduction of dysuria was more significative in the D + EE/D + EV groups rather than in the D group. Regarding tolerability, treatment associated side effects were reported by 16.2% patients. The most frequent one was uterine bleeding/spotting, significantly higher in the D + EV group.

**Conclusion:**

Dienogest alone or associated with estrogens (EE/EV) seems to be equally effective in reducing endometriotic lesions mean diameter. The reduction of dysmenorrhea was more significative when D was administered alone, while dysuria seems to improve more when D is associated with estrogens.

**Supplementary Information:**

The online version contains supplementary material available at 10.1007/s00404-023-07125-2.

## What does this study add to the clinical work

**Table:** 

To the best of our knowledge this is the first study comparing the effects of the three commercially available dienogest-containing in terms of pain symptoms and size of ovarian endometriomas changes. The results of our study could help clinicians to choose the correct therapy for patients with endometriosis, in the perspective of a long-term treatment.	

## Introduction

Endometriosis is a benign, chronic and inflammatory disease characterized by the presence of endometrium-like tissue outside the uterine cavity and is associated with pain and infertility [1]. The prevalence of the disease is estimated to be 5–10% of women of reproductive age [2, 3], and it may be found in 90% of women with pelvic pain [4]. Endometriosis-related pain symptoms have a negative impact on women’s quality of life and psychological wellbeing [1, 5, 6]. Ovarian endometriosis and in particular endometriotic cysts, also called endometriomas, represent the most frequent endometriotic lesion [7].

Both medical therapy and surgery are available for the treatment of ovarian endometriomas. They don’t offer a definitive cure for the disease, but evidence from observational studies suggests that they are both effective in reducing pain symptoms) [8]. Clinicians must provide patients with information about treatment related risks and benefits and consider women’s preference and objectives. When choosing medical treatment for endometriosis-associated pain, side effects, efficacy, costs and availability should be taken into account and discussed [1]. The main objectives of medical management are the improvement of pain symptoms and the prevention of postoperative recurrence, thereby eliminating the need for repeated surgery or prolonging the time between surgeries [9].

Regarding surgical treatment, several factors should be considered and discussed: patient’s age and ovarian reserve, fertility desire and treatment history, in particular previous ovarian surgery, pain symptoms and failure of medical treatment, size of endometrioma, bilaterality and suspicion for malignant involvement [10]. Surgical excision of ovarian endometriomas is burdened by the reduction in ovarian reserve and the high recurrence rate [11–13]. To avoid these risks, medical treatment may be the first choice. Among hormonal treatments, progestins with or without estrogens may be preferable due to their favorable safety, efficacy and tolerability and limited costs, especially in the perspective of a long-term therapy [1]. According to some studies they have also proved useful in the reduction of ovarian endometrioma size [14, 15].

Dienogest (D) is a semisynthetic progestin derived from 19-nortestosterone which binds progesterone receptors, blocking gonadotropin secretion. It also has a local antiproliferative and anti-inflammatory effect on endometriosis lesions and is effective in the reduction of pain symptoms with a favorable tolerability profile [16, 17]. Dienogest can be administered alone or in association with estrogens in two therapeutic formulations: ethinylestradiol (0.03 mg) and dienogest 2 mg in biphasic formulation, or estradiol valerate and dienogest in quadriphasic formulation. The main differences between the two estroprogestin (EP) formulations are the different metabolic impact, the thromboembolic risk and side effects related to the estrogen’s component [18–20].

Previous studies investigated the effect of these therapies in women with endometriosis [21–24]. Nevertheless, the comparison between the tree formulations has never been reported in literature.

The aim of our study is to compare the effects of the three commercially available dienogest-containing therapies (dienogest 2 mg alone, dienogest 2 mg combined with ethinylestradiol 0.03 mg or combined with estradiol valerate 1–3 mg) in terms of pain symptoms and size of ovarian endometriomas changes.

Secondary outcomes are:

- Comparison in terms of symptoms variations and endometriotic lesion reduction respectively between cyclic and continuous EP regimens and between D and continuous EP regimen
- Treatment tolerability
- Comparison in terms of changes in size of endometriotic nodules.

## Materials and methods

This retrospective study included 297 patients in reproductive age who referred to our Center from January 2017 to June 2021. Inclusion criteria were: (a) age between 18 and 50 years; (b) ultrasound diagnosis of ovarian endometrioma (> 10 mm in mean diameter) with or without deep infiltrating endometriosis or adenomyosis [24, 25]; (c) the presence of at least one of the following pain symptoms (Numeric Rating Scale > 0) [26]: dysmenorrhea, chronic pelvic pain, dyspareunia; (d) medical therapy for at least 12 months with dienogest 2 mg (D), or ethinylestradiol 0.03 mg/dienogest 2 mg (D + EE) or estradiol valerate 1–3 mg/dienogest 2 mg (D + EV). Exclusion criteria were: postmenopausal status, ongoing pregnancy or actual pregnancy desire, medical therapy in the 3 months before enrollment.

We retrospectively reviewed data from our clinical records. As in our daily practice, women were evaluated at baseline visit (V1), when therapy was prescribed, and after 6 and 12 months of therapy (follow-up visits V2 and V3, respectively). According to the therapy prescribed at V1, women were divided into three groups: [1] the first group received Dienogest 2 mg/day (D group); (2) the second group received ethinylestradiol 0.03 mg and dienogest 2 mg/day (D + EE group); (3) the third group received estradiol valerate 1–3 mg and dienogest 2 mg/ day (D + EV group). The type of administration (cyclic regimen or continuous regimen) for the D + EV and D + EE groups was recorded.

Medical history, detailed gynecological examination, transvaginal and transabdominal ultrasound were recorded in all women at each visit. Ultrasound examination was performed by sonographers experienced in endometriosis, making a subjective evaluation of grayscale and Doppler ultrasound “pattern recognition”: a “typical” ovarian endometrioma was diagnosed when a unilocular cyst with ultrasound features of regular wall, ‘ground glass’ echogenicity of the cyst content and poor capsular vascularization at Power Doppler was observed [27]. To assess the size of ovarian endometriomas, the three diameters (longitudinal, transverse, and antero-posterior) were measured and the mean diameter was then calculated (d1 + d2 + d3/3).

Data on demographic and clinical characteristics of the participants were collected: age, body mass index, parity, mean diameter of the cyst, presence of adenomyosis, presence of posterior nodule, presence of anterior endometriosis. Anatomic locations of endometriotic lesions at ultrasound were described according to IDEA consensus [25]. During visits, women were asked to rank endometriosis related symptoms (dysmenorrhea, chronic pelvic pain, dyspareunia) using a numerical Numerical Rating Scale (NRS) from 0 (absence of pain) to 10 (“the maximum pain you could imagine”) [26]. At each follow-up visits (V2 and V3), women were also asked to report any side effects related to the treatment (e.g.: weight gain, mood disorders, loss of libido, headache, nausea, acne, hair loss, breast tenderness, vaginal dryness, uterine bleeding including spotting).

### Statistical analysis

Numerical variables were summarized as mean ± standard deviation; categorical variables were summarized as frequencies and percentages. To investigate the presence of systematic differences in change scores and sizes after 12 months of follow-up between patients treated with dienogest alone and patients treated with dienogest in combination with ethinylestradiol (EE) or estradiol valerate (EV), we performed a linear regression analysis with heteroskedasticity-consistent standard errors, including treatment as a binary covariate in the models. To control for potential differences in baseline scores and sizes, baseline figures were also included in the models as continuous covariates. For illustrative purposes, a multilevel mixed-effects linear regression analysis was performed to investigate the course of symptoms and lesion sizes over the entire follow-up period (including the third and last visit), with random intercepts for each patient. Time was treated as a categorical covariate, which resulted in the inclusion of 2 dummy variables in the model, to assess the presence of nonlinear time trends. More specifically, we modelled each outcome as a function of time-by-therapy interactions in order to investigate the presence of divergent trajectories over time between the 2 study groups; predicted means with their 95% confidence intervals (CIs) resulting from multilevel modelling were then displayed using line charts. All analyses described above were replicated on the subsample of patients in treatment with D plus EE or EV. First, we compared EE and EV; second, we compared continuous and cyclic estrogen regimen. Lastly, the same analyses were performed to examine differences in symptoms according to presence or absence of adenomyosis. All analyses were carried out using Stata software, version 15 (StataCorp, 2017, Stata Statistical Software: Release 15, College Station, Texas, USA: Stata Corp LP). The significance level was set at 5%, and all tests were 2-sided.

## Results

We eventually included un our study 297 patients: 156 patients in the D group, 58 patients in the D + EE group and 83 patients in the D + EV group. The demographic and clinical characteristics are reported in Table 1.

**Table 1:** Baseline characteristics of the study sample, overall and by treatment

Variable	All (*n* = 297)	Treatment			*P* value
		Dienogest alone	Dienogest + EE	Dienogest + EV	
		(*n* = 156)	(*n* = 58)	(*n* = 83)	
Age, years	33.6 ± 7.9	35.5 ± 7.6	28.9 ± 6.6	33.5 ± 8.2	< 0.001***
Body mass index, kg/m^2^	22.7 ± 4.3	23.4 ± 5.0	22.0 ± 3.8	22.0 ± 2.8	0.061
Endometrial cyst, mm	23.5 ± 15.1	26.3 ± 14.8	22.7 ± 13.8	18.7 ± 15.3	< 0.001
Adenomyosis	158 (53.2%)	84 (53.9%)	35 (60.3%)	39 (47.0%)	0.286
Posterior nodule	118 (39.7%)	72 (46.2%)	22 (37.9%)	24 (28.9%)	0.033*
Anterior endometriosis	0 (0.0%)	0 (0.0%)	0 (0.0%)	0 (0.0%)	

Values are mean ± standard deviation or *n* (%). *P* values were obtained with the chi-squared test, Kruskal–Wallis test or analysis of variance, where appropriate. Mean diameter of cysts and nodules is the average of length (longitudinal diameter), width (transverse diameter) and antero-posterior diameter; in case of > 1 lesion, the largest one was analyzed

*EE* ethinylestradiol, *EV* estradiol valerate

*P ≤ 0.05

**P ≤ 0.01

***P ≤ 0.001

### Effects on symptoms

Regarding symptoms, when comparing women treated with D and women treated with D combined with estrogens, a significant decrease in terms of dysuria was detected in the EP groups (− 0.30 in D + EE/EV groups) rather than the D group (0.08 in D group) between baseline and second follow-up evaluation V3 (Table 2). In contrast, the reduction of the dysmenorrhea was more significative in D group (− 2.63 vs − 2.04 in D + EE/EV groups). The statistically significant reduction in the dysmenorrhea in D group is also confirmed when comparing D group with continuous administration in the D + EE/EV groups (− 1.73 in D + EE/EV groups VS − 2.63 in D group, adjusted Δ for baseline NRS scores 0.96, *P* ≤ 0.05), as well as the significant reduction of dysuria in the EP groups (− 0.45 in D + EE/EV groups vs 0.08 in D group, adjusted Δ for baseline NRS scores − 0.30, *P* ≤ 0.05). Furthermore, no significant difference regarding symptoms was found when comparing group D alone with group D + EE/EV in continuous regimen (Supplementary Material 1, S1).

**Table 2:** Treatment-related changes in symptoms and in lesion mean diameter between the baseline (V1) and 2nd follow-up evaluation (V3, 12 months apart), and differences in changes between treatments (D + EE/EV vs D), both crude (Δ) and adjusted for baseline NRS scores and sizes (adj. Δ)

	Dienogest + EE/EV	Dienogest Alone		
Variable	(*n* = 141)	(*n* = 156)	Δ	Adj. Δ
	Mean (95% CI)	Mean (95% CI)		
Dyspareunia, NRS	− 0.79***	− 0.69**	− 0.10	− 0.02
	(− 1.27, − 0.32)	(− 1.15, − 0.24)		
Chronic pelvic pain, NRS	− 0.46	− 0.72**	0.26	0.01
	(− 0.94, 0.02)	(− 1.25, − 0.18)		
Dysmenorrhea	− 2.04***	− 2.63***	0.59	0.77**
	(− 2.71, − 1.38)	(− 3.29, − 1.96)		
Dysuria w/menses, NRS	− 0.86***	0.00	− 0.86^***^	− 0.23*
	(− 1.25, − 0.47)	(− 0.16, 0.16)		
Dysuria, NRS	− 0.30**	0.08	− 0.38^**^	− 0.23*
	(− 0.53, − 0.07)	(− 0.05, 0.21)		
Dyschezia w/menses, NRS	− 0.38*	− 0.44*	0.05	− 0.09
	(− 0.69, − 0.08)	(− 0.85, − 0.02)		
Dyschezia, NRS	0.06	− 0.08	0.14	− 0.12
	(− 0.22, 0.34)	(− 0.45, 0.28)		
Endometrial cyst, mm	− 5.16***	− 6.11***	0.95	− 1.13
	(− 7.02, − 3.30)	(− 7.75, − 4.47)		
Posterior nodule, mm	0.17	− 0.35	0.53	− 0.03
	(− 0.83, 1.18)	(− 1.51, 0.80)		

Mean diameter of cysts and nodules is the average of length (longitudinal diameter), width (transverse diameter) and antero-posterior diameter; in case of > 1 lesion, the largest one was analyzed

*EE* ethinylestradiol, *EV* estradiol valerate, *CI* confidence interval, *NRS* numeric rating scale

*P ≤ 0.05

**P ≤ 0.01

***P ≤ 0.001

When comparing cyclic and continuous administration of the EP groups (D + EE and D + EV), there were no statistically significant differences in terms of symptoms changes (Table 3).

**Table 3:** Treatment-related changes in symptoms and in lesion mean diameter between the baseline (V1) and 2nd follow-up evaluation (V3, 12 months apart), and differences in changes between EP treatments (D + EV vs D + EE), both crude (Δ) and adjusted for baseline NRS scores and sizes (adj. Δ)

	Dienogest + EV	Dienogest + EE		
Variable	(*n* = 83)	(*n* = 58)	Δ	Adj. Δ
	Mean (95% CI)	Mean (95% CI)		
Dyspareunia, NRS	− 1.14***	− 0.29	− 0.85	− 0.68
	(− 1.67, − 0.62)	(− 1.15, 0.57)		
Chronic pelvic pain, NRS	0.24	− 1.47***	1.71***	0.69
	(− 0.30, 0.78)	(− 2.28, − 0.65)		
Dysmenorrhea	− 1.75***	− 2.47***	0.72	0.37
	(− 2.65, − 0.84)	(− 3.43, − 1.50)		
Dysuria w/menses, NRS	− 1.39***	− 0.10	− 1.29***	0.00
	(− 2.03, − 0.75)	(− 0.25, 0.04)		
Dysuria, NRS	− 0.51**	0.00	− 0.51**	0.00
	(− 0.89, − 0.12)	(0.00, 0.00)		
Dyschezia w/menses, NRS	− 0.34	− 0.45	0.11	0.19
	(− 0.75, 0.08)	(− 0.90, 0.01)		
Dyschezia, NRS	0.10	0.00	0.10	0.27
	(− 0.31, 0.50)	(− 0.37, 0.37)		
Endometrial cyst, mm	− 4.52***	− 6.08***	1.56	0.04
	(− 7.07, − 1.96)	(− 8.78, − 3.38)		
Posterior nodule, mm	0.34	− 0.07	0.41	0.05
	(− 0.47, 1.16)	(− 2.27, 2.12)		

Mean diameter of cysts and nodules is the average of length (longitudinal diameter), width (transverse diameter) and antero-posterior diameter; in case of > 1 lesion, the largest one was analyzed

*EV* estradiol valerate, *EE* ethinylestradiol, *CI* confidence interval, *NRS* numeric rating scale

*P ≤ 0.05

**P ≤ 0.01;

***P ≤ 0.001;

### Effects on lesions

The differences in lesion mean diameter between baseline and second follow-up examination (V3) in women treated with D alone or D combined with estrogens (D + EE/EV) are shown in Table 2. There were no statistically significant differences between the D group and the EP groups in lesion mean diameter variations (both endometriomas and endometriotic nodules).

Regarding EPs, no differences in lesion mean diameter were found either with regard to the type of estrogen administered (D + EE vs D + EV) (Table 3) or the administration regimen (continuous vs cyclic) (Table 4) (ovarian endometriomas − 5.64 vs − 6.11, Adjusted Δ for baseline sizes − 2.01; endometrial nodules 0.006 vs − 0.35, Adjusted Δ for baseline sizes − 0.19). Moreover, when comparing D alone with continuous administration of EPs (D + EE/EV), no differences in lesion mean diameter were reported (Supplemental Material S1).

**Table 4:** Treatment-related changes in symptoms and in lesion mean diameter between the baseline (V1) and 2nd follow-up evaluation (V3, 12 months apart), and differences in changes between cyclic and continuous EP treatments, both crude (Δ) and adjusted for baseline NRS scores and sizes (adj. Δ)

	Dienogest + cyclic	Dienogest + contin		
Variable	EE/EV (*n* = 86)	EE/EV (*n* = 55)	Δ	Adj. Δ
	Mean (95% CI)	Mean (95% CI)		
Dyspareunia, NRS	− 0.73**	− 0.89*	0.16	0.12
	(− 1.29, − 0.18)	(− 1.75, − 0.04)		
Chronic pelvic pain, NRS	− 0.62	− 0.22	− 0.40	− 0.32
	(− 1.27, 0.04)	(− 0.90, 0.46)		
Dysmenorrhea	− 2.24***	− 1.73***	− 0.52	− 0.31
	(− 3.00, − 1.49)	(− 2.97, –0.49)		
Dysuria w/menses, NRS	− 0.92***	− 0.76	− 0.15	0.00
	(− 1.40, − 0.43)	(− 1.43, − 0.10)		
Dysuria, NRS	− 0.20	− 0.45	0.26	0.00
	(− 0.43, 0.04)	(− 0.91, 0.00)		
Dyschezia w/menses, NRS	− 0.52*	− 0.16	–0.36	0.01
	(− 0.98, − 0.06)	(− 0.48, 0.16)		
Dyschezia, NRS	0.03	0.09	–0.06	0.08
	(− 0.36, 0.43)	(− 0.27, 0.45)		
Endometrial cyst, mm	− 4.85***	− 5.64**	0.79	1.04
	(− 6.97, − 2.73)	(− 9.11, − 2.17)		
Posterior nodule, mm	0.25	0.06	0.19	0.30
	(− 0.81, 1.30)	(− 1.96, 2.08)		

Mean diameter of cysts and nodules is the average of length (longitudinal diameter), width (transverse diameter) and antero-posterior diameter; in case of > 1 lesion, the largest one was analyzed

*EE* ethinylestradiol, *EV* estradiol valerate, *CI* confidence interval, *NRS* numeric rating scale

*P ≤ 0.05

**P ≤ 0.01

***P ≤ 0.001

### Tolerability

Regarding tolerability, treatment associated side effects were reported by 16.2% of women, of which 7.1% treated with D, 6.7% treated with D + EV and 2.4% treated with D + EE. Side effects during treatment are reported in Table 5. The most frequent side effect was uterine bleeding/spotting. Spotting was significantly more frequent in group D + EV than in the other two groups (*P* = 0.04) and was reported in particular by women who assumed treatment in continuous administration (4 of 28 patients who assumed D + EV continuously, 14.3%). No significant differences were found in other side effects between the three groups.

**Table 5:** Distribution of side effects, overall and by treatment. Values are counts (percentages)

Side effect	All (*n* = 297)	Treatment			Exact *P* value
		Dienogest alone	Dienogest + EV	Dienogest + EE	
		(*n* = 156)	(*n* = 83)	(*n* = 58)	
Any	48 (16.2)	21 (13.5)	20 (24.1)	7 (12.1)	0.07
Spotting	14 (4.7)	5 (3.2)	8 (9.6)	1 (1.7)	0.04*
Headache	12 (4.0)	7 (4.5)	3 (3.6)	2 (3.4)	0.92
Mood swing	10 (3.4)	7 (4.5)	2 (2.4)	1 (1.7)	0.52
Weight gain	9 (3.0)	3 (1.9)	5 (6.0)	1 (1.7)	0.17
Bloating	8 (2.7)	2 (1.3)	4 (4.8)	2 (3.4)	0.25
Loss of libido	5 (1.7)	3 (1.9)	2 (2.4)	0 (0.0)	0.52
Vaginal dryness	3 (1.0)	3 (1.9)	0 (0.0)	0 (0.0)	0.25
Hair loss	1 (0.3)	1 (0.6)	0 (0.0)	0 (0.0)	0.64
Tachycardia	1 (0.3)	0 (0.0)	1 (1.2)	0 (0.0)	0.27
Double vision	1 (0.3)	0 (0.0)	1 (1.2)	0 (0.0)	0.27

*EE* ethinylestradiol, *EV* estradiol valerate

*P ≤ 0.05

**P ≤ 0.01

***P ≤ 0.001

## Discussion

In this retrospective study we analyzed the impact in terms of symptoms and endometriotic lesion mean diameter variations in patients treated with dienogest alone or dienogest combined with estrogens (D + EE and D + EV).

### Effects on lesions and symptoms

Regarding symptoms, as previously demonstrated in literature [16], a statistically significant reduction in the severity of dysmenorrhea associated with D-only therapy was found in comparison with dienogest combined with estrogens (both EE and EV*)* after 12 months of therapy [28, 29]. The greater reduction in dysmenorrhea may likely be related to the induction of amenorrhea together with the antiproliferative and anti-inflammatory effect of D. This result is in line with the prospective study by Caruso et al.: in their cohort of 44 patients, they reported amenorrhea in 88.3% of patients after 24 months of D-only therapy, with a significant decrease of dysmenorrhea and pelvic pain [23].

Moreover, in our study population the improvement of dysuria was greater in EP groups rather than the D group. This finding may be due to the trophic action of estrogens on the urethral mucosa [30] and to lower estrogen levels induced by dienogest [31].

Our data showed that there are no differences between oral administration of D and D + EE or D + EV in terms of endometriotic lesion mean diameter changes. The effect of dienogest on the size of ovarian endometriomas has already been studied in Literature showing that the hypoestrogenic state induced by the therapy reduces inflammation and proliferation of ectopic endometrium-like tissue, leading to a possible decrease in lesion size [29, 32]. Different results regarding lesions variations are reported by other studies with smaller samples comparing D alone or associated with EE in women with endometriosis. In a retrospective observational study conducted on women with ovarian endometriomas [21], Xholli and colleagues reported a reduction in the size of endometriomas in patients treated with D alone (*n* = 34) and with D + EE (*n* = 36) after 12 months, but the reduction was greater in patients treated with D alone. On the other hand, a prospective study conducted on 81 women treated with D or D + EE detected a significant decrease in endometrioma’s volume only in the group treated with D after 3 and 6 months of follow up [22].

### Tolerability

Regarding drug tolerability, uterine bleeding/spotting was reported more frequently in the D + EV group, especially when administered in a continuous regimen. Our data suggest that when D + EV is chosen, a cyclic administration may be preferable than continuous. Considering that endometriosis is a chronic disease, in the perspective of a long-term treatment, tolerability and consequent compliance to treatment are fundamental aspects of medical management of women with endometriosis.

### Strengths and limitations

The main limitations of our study are its retrospective nature and the absence of a control group. Although the lack of histological confirmation, ultrasound has shown a high diagnostic accuracy for endometriosis in particular if performed by expert sonographers as in our study [25]. Regarding patients’ characteristics, we found a statistically significant difference regarding the age of patients: women treated with D + EE therapy were younger than the rest of the study population. This may be due to the fact that EPs are usually prescribed to adolescents and younger patients rather than progestogen alone. The effect of dienogest on bone mineral density (BMD) is still controversial in the Literature and, according to some studies, it may reduce BMD [33], therefore especially in adolescent patients the choice of combining dienogest with an estrogen seems reasonable. In addition, we found a significant difference in the size of ovarian endometriomas at baseline visit; nevertheless, our primary outcome was the cysts size changes over time, therefore this finding was not a limitation for the analysis.

This study has also some strengths: to the best of our knowledge, we reported the largest cohort of patients with ovarian endometriomas assuming dienogest-based hormonal therapies and investigate for the first-time differences among these three therapeutic options. Another strength is represented by the long-term follow-up.

## Conclusions

In conclusion, the comparison of Dienogest alone or associated with estrogens (EE or EV) showed no difference in terms of lesion size variation and pain symptoms in women with ovarian endometriomas, except for dysmenorrhea, which seems to benefit more from progestin-only therapy, and dysuria, which improves more with EP treatment. In the light of our results, we believe that clinicians should consider efficacy of the different therapies on symptoms relief, together with tolerability, metabolic impact and thromboembolic risk of each hormonal treatment, as well as women’s age, comorbidities and preference. Balancing all these aspects will let clinicians choose the right treatment for each woman, in the perspective of a long-term treatment, improving adherence to treatment and consequently reducing the risk of disease progression over time.

## Supplementary Information

Below is the link to the electronic supplementary material.

Supplementary file1 (DOCX 37 KB): S1. Treatment-related changes in symptoms and in lesion mean diameter between the baseline (V1) and 2nd follow-up evaluation (V3, 12 months apart), and differences in changes between treatments (D+EE/EV vs D), both crude (Δ) and adjusted for baseline NRS scores and sizes (adj. Δ). S2. Line plots showing mean diameter of endometrial cyst and posterior nodule during follow-up (baseline V1, 1st follow-up examination V2 6 months apart, 2nd follow-up evaluation V3 12 months apart) by therapy (dienogest alone [DNG] and in combination with ethinylestradiol or estradiol valerate [DNG + EE/EV]). Mean estimates resulting from multilevel mixed-effects analysis are presented along with 95% confidence intervals.

## Data Availability

The data that support the findings of this study are available on request from the corresponding author, [B.O.]. The data are not publicly available due to their containing information that could compromise the privacy of research participants.
