# Supplementary material for: Dienogest alone or dienogest combined with estrogens in the treatment of ovarian endometriomas, that is the question. A retrospective cohort study
Source: Arch Gynecol Obstet. 2023 Jul 12;308(4):1341–9. doi: 10.1007/s00404-023-07125-2 (PMC10435622; doi:10.1007/s00404-023-07125-2)
Supplement: Supplementary file 1 — Supplementary file1 (DOCX 37 KB): S1. Treatment-related changes in symptoms and in lesion mean diameter between the baseline (V1) and 2nd follow-up evaluation (V3, 12 months apart), and differences in changes between treatments (D+EE/EV vs D), both crude (Δ) and adjusted for baseline NRS scores and sizes (adj. Δ). S2. Line plots showing mean diameter of endometrial cyst and posterior nodule during follow-up (baseline V1, 1st follow-up examination V2 6 months apart, 2nd follow-up evaluation V3 12 months apart) by therapy (dienogest alone [DNG] and in combination with ethinylestradiol or estradiol valerate [DNG + EE/EV]). Mean estimates resulting from multilevel mixed-effects analysis are presented along with 95% confidence intervals. [file 404_2023_7125_MOESM1_ESM.docx]

**SUPPLEMENTARY MATERIAL**

**S1.** Treatment-related changes in symptoms and in lesion mean diameter between the baseline (V1) and 2^nd^ follow-up evaluation (V3, 12 months apart), and differences in changes between treatments (D+EE/EV vs D), both crude (Δ) and adjusted for baseline NRS scores and sizes (adj. Δ).

|  | Dienogest + contin. | Dienogest Alone |  |  |
| --- | --- | --- | --- | --- |
| Variable | EE/EV (*n* = 55) | (*n* = 156) | Δ | Adj. Δ |
|  | Mean (95% CI) | Mean (95% CI) |  |  |
| Dyspareunia, NRS | –0.89^*^ | –0.69^**^ | –0.20 | –0.09 |
|  | (–1.74, –0.04) | (–1.15, –0.24) |  |  |
| Chronic pelvic pain, NRS | –0.22 | –0.72^**^ | 0.50 | 0.20 |
|  | (–0.90, 0.46) | (–1.26, –0.18) |  |  |
| Dysmenorrhea | –1.73^**^ | –2.63^***^ | 0.90 | 0.96^*^ |
|  | (–2.96, –0.49) | (–3.30, –1.96) |  |  |
| Dysuria, NRS | –0.45^*^ | 0.08 | –0.54^*^ | –0.30^*^ |
|  | (–0.91, 0.00) | (–0.05, 0.21) |  |  |
| Dyschezia w/ menses, NRS | –0.16 | –0.44^*^ | 0.27 | –0.13 |
|  | (–0.48, 0.15) | (–0.85, –0.02) |  |  |
| Endometrial cyst, mm | –5.64^***^ | –6.11^***^ | 0.47 | –2.01 |
|  | (–9.09, –2.19) | (–7.75, –4.46) |  |  |
| Posterior nodule, mm | 0.06 | –0.35 | 0.41 | –0.19 |
|  | (–1.95, 2.07) | (–1.52, 0.81) |  |  |

^***^*P* value ≤0.001; ^**^*P* value ≤0.01; ^*^*P* value ≤0.05.

*Notes:* Mean diameter of cysts and nodules is the average of length (longitudinal diameter), width (transverse diameter) and antero-posterior diameter; in case of >1 lesion, the largest one was analyzed.

*Abbreviations:* EE, ethinylestradiol; EV, estradiol valerate; CI, confidence interval; NRS, Numeric Rating Scale.

**S2.** Line plots showing mean diameter of endometrial cyst and posterior nodule during follow-up (baseline V1, 1^st^ follow-up examination V2 6 months apart, 2^nd^ follow-up evaluation V3 12 months apart) by therapy (dienogest alone [DNG] and in combination with ethinylestradiol or estradiol valerate [DNG + EE/EV]). Mean estimates resulting from multilevel mixed-effects analysis are presented along with 95% confidence intervals.

*Notes:* Mean diameter is the average of length (longitudinal diameter), width (transverse diameter) and antero-posterior diameter; in case of >1 lesion, the largest one was analyzed.
